# The relationship of benign prostatic hyperplasia's symptoms severity with the risk of developing atrial fibrillation

**DOI:** 10.1002/joa3.12684

**Published:** 2022-02-09

**Authors:** Ajar Koçak, Cem Şenol, Ayhan Coşgun, Ferhat Eyyupkoca, Onur Yıldırım

**Affiliations:** ^1^ Department of Cardiology Sincan State Hospital Ankara Turkey; ^2^ Department of Urology Sincan State Hospital Ankara Turkey

**Keywords:** atrial electromechanical delay, atrial fibrillation, benign prostatic hyperplasia, P‐wave dispersion, tissue doppler imaging

## Abstract

**Background:**

Attention is drawn to the increased incidence of atrial fibrillation (AF) in benign prostatic hyperplasia (BPH) patients recently. Early predicting of AF in these patients can help in decreasing its clinical consequences. The aim of our study is to determine the association between BPH symptoms and AF predictors atrial electromechanical delay (AEMD) and the P‐wave dispersion (PWD).

**Methods:**

218 healthy individuals recently diagnosed with BPH were assigned into three groups according to symptoms severity using the International prostate symptom score (IPSS) questionnaire. The first group with mild symptoms (IPSS score between 0 and 7, *n* = 78), the second group with moderate symptoms (IPSS score between 8 and 19, *n* = 86), and the third group with severe symptoms (IPSS score between 20 and 35, *n* = 54). PWD and AEMD calculations were performed for all participants.

**Results:**

There were statistically significant differences between the three groups in terms of AEMD and PWD (*p* < .01 and *p* < .01, respectively). In all three study groups, a significant positive correlation was observed between IPSS questionnaire scores and both AEMD and PWD (for AEMD *r* = .29, *p* = .013 and for PWD *r* = .27, *p* = .017). On the other hand, there were significant differences between the three groups in terms of the inflammatory markers C‐reactive protein (CRP) and fibrinogen (*p* < .01 and *p* < .01, respectively) and in terms of serum testosterone levels (*p* < .01).

**Conclusions:**

We concluded that periodic evaluation of patients with BPH in terms of symptoms severity can be helpful not only from urological aspect, but also in the early prediction of possible serious cardiovascular morbidity and mortality.

## Graphical abstract

In this study, we aimed to find simple and cheap methods to help clinicians predict the risk of atrial fibrillation in benign prostatic hyperplasia patients. We evaluated electrocardiographic and echocardiographic parameters that are considered as predictors of atrial fibrillation in these patients and found that there is a direct correlation between these parameters and the severity of prostatic hyperplasia symptoms. We concluded that periodic evaluation of patients with benign prostatic hyperplasia in terms of symptoms severity can be helpful not only from urological aspect, but also in the early prediction of possible serious cardiovascular morbidity and mortality.

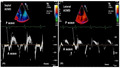

## 1 INTRODUCTION

Benign prostatic hyperplasia (BPH) is the most common disease of the prostate gland. Its incidence increases with age and can seriously affect the patient's quality of life.^1^ The majority of BPH patients consists of old males and among them there is an increased risk of cardiovascular events (CVE) which may be due to the increased incidence of cardiovascular risk factors such as hypertension (HT), diabetes mellitus (DM), and dyslipidemia (DLP), also because of the increased incidence of serious cardiac arrhythmias like atrial fibrillation (AF).^2^, ^3^, ^4^ The increased incidence of AF in PBH patients was observed in a retrospective study including 15 760 participants. In the study, while the annual incidence of AF was 4.77% in the BPH patient group, this rate was 3.76% in the control group.^5^ An important pathophysiological factor that plays role in devolving BPH is chronic inflammation which can also increase the risk of AF.^1^, ^6^ Another factor is serum testosterone levels, as it is known that low testosterone levels increase prostatic inflammation and hence can aggravate BPH symptoms,^7^, ^8^ and recently it has been shown that low testosterone levels are also associated with increased risk of atrial fibrillation in male patients.^9^

Atrial fibrillation is a very common rhythm disorder and a major contributor to morbidity especially among the old population due to associated strokes, heart failure, and quality of life impairment.^10^ Early detection of AF can be very useful in decreasing its clinical consequences, and this currently gained wide interest among the cardiovascular community. Several diagnostic methods were used to identify individuals with high risk of developing AF, among which are the atrial electromechanical delay time (AEMD) and the P‐wave dispersion (PWD).^11^, ^12^ P‐wave dispersion is obtained by subtracting the shortest P‐wave duration from the longest P‐wave duration on the standard 12‐lead electrocardiogram (ECG). AEMD is defined as the temporal delay between the detected onset of electrical activity in the atrium (the start point of the P wave on surface ECG) and the realization of force in the atrial myocardium (the start point of the A wave detected by tissue doppler imaging on echocardiography). Although AEMD is a simple non‐invasive diagnostic tool, it is considered as an indicator of atrial conduction heterogeneity and correlates well with invasive electrophysiological findings.^13^

The aim of our study is to understand the relationship between BPH symptoms severity using the International prostate symptom score (IPSS) questionnaire and AF predictors such as AEMD and PWD to help find methods for early detection of AF in BPH patients with no other comorbidities. We also evaluated the possible pathophysiological factors that can play role in both clinical conditions such as chronic inflammation and serum testosterone levels.

## 2 MATERIALS AND METHODS

### 2.1 Study population

In all, 218 healthy individuals recently diagnosed with BPH in our hospital were included in the study between August 2020 and July 2021. Patients were assigned into three groups according to IPSS questionnaire, the first group included 78 patients with mild symptoms (IPSS score between 0 and 7), the second group included 86 patients with moderate symptoms (IPSS score between 8 and 19), and the third group included 54 patients with severe symptoms (IPSS score between 20 and 5).^14^ Laboratory test results dated to the time of BPH diagnosis were obtained from patients' medical records.

### 2.2 Exclusion criteria

Having any disease that increases the burden of the heart, such as anemia or electrolyte disorder, having an obstructive lung disease, having a liver or kidney disease, having abnormal thyroid function test, having any abnormality that indicate a cardiac problem on transthoracic echocardiogram (TTE) like reduced ejection fraction (EF), hypertrophy or dilatation in any heart cavity, the presence of a right or left bundle branch block on the ECG, previous cerebrovascular accident, or a disease that may impair autonomic functions such as Parkinson's and patients diagnosed with cancer.

### 2.3 Electrocardiogram

The 12‐lead ECG data were taken with the patients in a supine position and after a 5‐min rest. While ECG recordings were taken, the paper speed was 25 mm/sec, and its amplitude was 10 mm/mV. (CardiofaxV model 9320, Nihon Kohden). ECGs were measured manually using a magnifying glass (TorQ, 150 mm Digital Caliper LCD). All ECGs were evaluated by a cardiologist blinded to patients' clinical information. The P‐wave duration was defined as the time elapsed from the beginning of the P wave to the end. PWD in the standard 12‐lead ECG was defined and calculated as subtracting the shortest P duration (Pmin) from the longest P duration (Pmax) (PWD = Pmax−Pmin) occurring in any of the 12 leads.

### 2.4 Transthoracic echocardiogram

**FIGURE 1:**
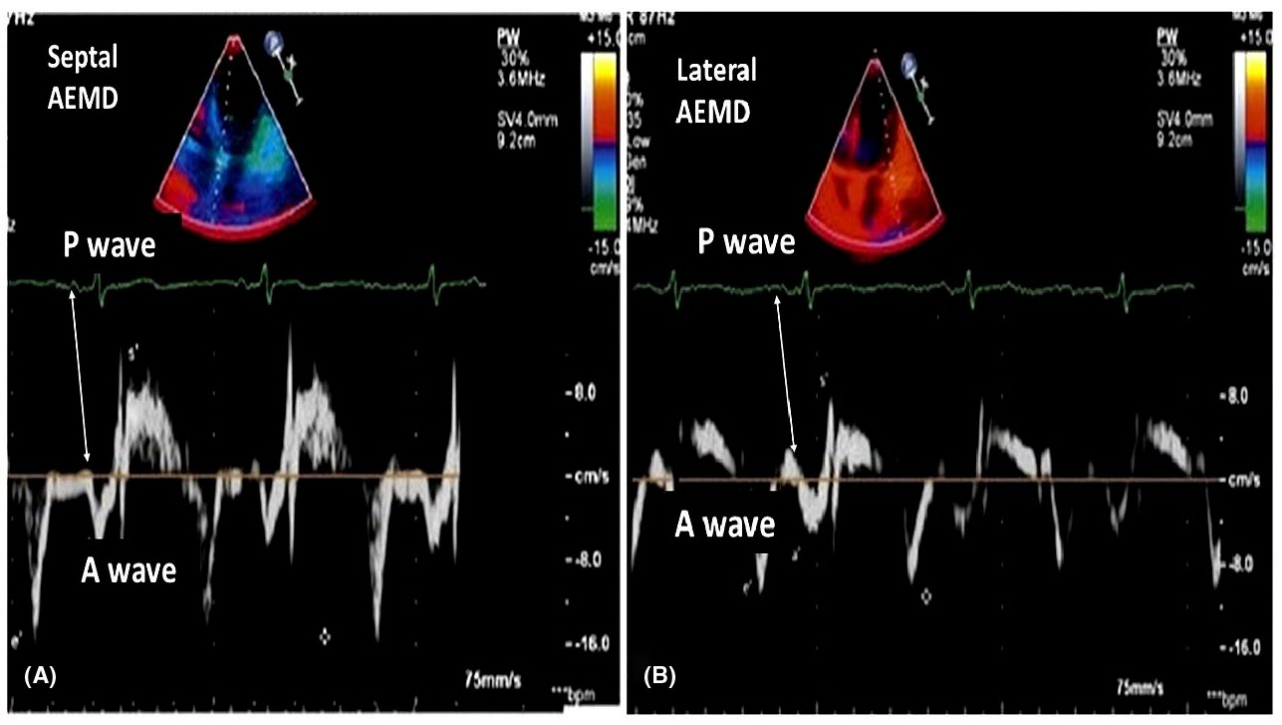
Image of echocardiographic doppler signals using tissue doppler imaging in apical four‐chamber view showing an example of atrial electromechanical delay time measurement at the septal and lateral positions of the mitral annular plane

Each BPH patient in the three groups was evaluated by TTE. The procedure was performed by a cardiologist blinded to patients' clinical information. The operator used Philips HD11XE, 2012 Netherland device for the procedure. Routine TTE analysis was performed according to the American Echocardiography Association guidelines.^15^ Digital images containing at least three heartbeats were recorded and evaluated. Tissue Doppler Imaging (TDI) was performed with pulse‐wave doppler. TDI examination was done at the apical four‐chamber view. During TDI examination, the cursor was placed in the lateral and septal positions of valves annuluses, respectively, and measurements were taken. Averages of three consecutive measurements taken from each valve annulus were noted. Then AEMD was calculated by taking the average of all measurements. AEMD was defined as the time from the start of the P wave in the D2 derivation to the start of the A wave in the TDI image, and the measurement was made and recorded in milliseconds (Figure 1). To assess interobserver variability of both PWD and AEMD, 50 participants were randomly selected and Bland–Altman plot analysis was performed. It was found that measurements were similar and statistically comparable with each other.

### 2.5 Statistics

Continuous variables were defined as mean ± standard deviation. Categorical variables were defined as percentages. Student's t‐test was used to compare continuous variables and chi‐square test was used for categorical variables. To compare the values of three separate groups, the ANOVA independent test was used. Z test was used to compare ratios. Pearson's correlation test was used to determine the correlation between the variables. *p* < .05 was considered statistically significant. Statistical analysis was performed using SPSS version 20.0 (IBM Co.,).

### 2.6 Ethics statement

All procedures, including the informed consent process, were conducted in accordance with the ethical standards of the National Health and Medical Research Council of Turkey and with the Helsinki Declaration of 1964 (as revised in 2013). The study was approved by the ethical committee of Dr. Abdurrahman Yurtaslan Ankara Oncology Training and Research Hospital (No:2020–07/714).

## 3 RESULTS

There were no statistically significant differences between the three groups in terms of the basal clinical and sociodemographic characteristics (Table 1).

**TABLE 1:** The basal clinical characteristics and sociodemographic properties of the study participants

Variable	Group 1	Group 2	Group 3	*p* value
Age, years	67.3 ± 5.3	68.1 ± 4.7	66.9 ± 4.4	.48
Basal HR, b/m	81.5 ± 7.4	82.7 ± 9.1	82.6 ± 8.8	.62
BMI, kg/m^2^	25.3 ± 3.2	24.9 ± 2.9	25.0 ± 4.1	.73
Systolic BP, mm Hg	122.5 ± 12.6	121.9 ± 11.3	119.4 ± 13.8	.34
Diastolic BP, mm Hg	77.4 ± 12.3	79.1 ± 11.5	78.3 ± 11.9	.65
LV mass, g	173.6 ± 31.7	171.7 ± 29.5	174.3 ± 27.2	.98
Hemoglobin, gr/dl	14.2 ± 1.7	14.3 ± 2.1	13.9 ± 1.6	.45
Basal sPAP, mm Hg	15.3 ± 3.1	14.7 ± 2.7	15.2 ± 4.3	.46
TC. mg/dl	171.8 ± 27.4	173.9 ± 31.7	175.3 ± 29.6	.79
LDL, mg/dl	135.8 ± 22.6	137.8 ± 31.9	141.4 ± 31.2	.46
Triglyceride, mg/dl	175.9 ± 35.7	169.5 ± 41.6	166.7 ± 33.6	.34
HDL, mg/dl	31.7 ± 4.8	33.1 ± 5.3	31.9 ± 4.7	.15
Calcium, mg/dl	9.3 ± 0.8	9.1 ± 0.7	9.4 ± 0.6	.34
Sodium, mEq/L	140.5 ± 1.4	140.9 ± 1.4	140.7 ± 1.2	.17
Potassium, mEq/L	4.1 ± 0.7	4.2 ± 0.5	4.3 ± 0.8	.22
TSH, mIU/L	3.3 ± 1.3	3.6 ± 0.9	3.4 ± 0.8	.17

Abbreviations: BMI, Body Mass Index; BP, Blood Pressure; LV, Left ventricle; TC, Total Cholesterol; LDL, Low‐Density Lipoprotein; HDL, High‐Density Lipoprotein; TSH, Thyroid Stimulating Hormone; sPAP, systolic pulmonary arterial pressure.

There were statistically significant differences between the three groups in terms of AEMD and PWD. Regarding the AMED durations, values were as following; group 1 = 29.5 ± 2.2, group 2 = 35.2 ± 3.7, and group 3 = 41.3 ± 4.4 (*p* < .01). And for the PWD values, group 1 = 32.5 ± 3.5, group 2 = 36.7 ± 5.2, and group 3 = 44.2 ± 7.6 (*p* < .01) (Table 2). A positive correlation was found between the IPSS score and both AEMD and PWD in all patients (for AEMD *r* = .29, *p* = .013 and for PWD *r* = .27, *p* = .017). When each group evaluated separately, the positive correlation was more significant in the third group with the highest IPSS score (for AEMD *r* = .33, *p* = .01 and for PWD *r* = .31, *p* = .02) than the other two groups in which correlation values were as follows, the second group (for AEMD *r* = .26, *p* = .01 and for PWD *r* = .29, *p* = .04) and the first group (for AEMD *r* = .24, *p* = .03 and for PWD *r* = .27, *p* = .03).

**TABLE 2:** The comparison table of Testosterone, PWD, AEMD, CRP, fibrinogen, and IPSS values of the study participants

Variables	Group 1	Group 2	Group 3	*P* value
IPSS score	3.4 ± 0.21	18.4 ± 2.56	28.2 ± 3.5	<.01
CRP, mg/dl	0.41 ± 0.03	2.3 ± 0.42	3.03 ± 0.74	<.01
Fibrinogen, mg/dl	214.1 ± 18.3	281.6 ± 25.3	313.8 ± 35.2	<.01
Testosterone, ng/ml	2.51 ± 0.14	2.46 ± 0.12	2.31 ± 0.19	<.01
PWD, ms	32.5 ± 3.5	36.7 ± 5.2	44.2 ± 7.6	<.01
AEMD, ms	29.5 ± 2.2	35.2 ± 3.7	41.3 ± 4.4	<.01

Abbreviations: AEMD, Atrial electromechanical delay time; CRP, C‐Reactive protein; IPSS, International Prostate Symptom Score; PWD, P‐wave Dispersion.

On the other hand, there were significant differences between the three groups in terms of the inflammatory markers C‐reactive protein (CRP) (Group 1 = 0.41 ± 0.03, group 2 = 2.3 ± 0.42, and group 3 = 3.03 ± 0.74 mg/dl, *p* < .01) and Fibrinogen (Group 1 = 214.1 ± 18.3, group 2 = 281.6 ± 25.3, and group 3 = 313.8 ± 35.2 mg/dl, *p* < .01) (Table 2). There were also a significant positive correlation between IPSS questionnaire score and CRP values in groups 2 and 3 (*r* = .22, *p* = .049 and *r* = .29, *p* = .01, respectively). There was no significant correlation between the IPSS questionnaire score and CRP values in group 1 (*r* = .11, *p* = .33). In addition, there were no significant correlations between IPSS questionnaire score and fibrinogen values in groups 1 and 2 (*r* = .13, *p* = .25 and *r* = .15, *p* = .18, respectively) but there was a significant positive correlation between the IPSS questionnaire score and fibrinogen values in group 3 (*r* = .25, *p* = .02).

Regarding testosterone levels, it was low in general and there were significant differences between the three groups as follows: group 1 = 2.51 ± 0.14, group 2 = 2.46 ± 0.12, and group 3 = 2.31 ± 0.19 ng/ml (*p* < .01) (Table 2). There were no significant correlations between IPSS questionnaire score and testosterone values in groups 1 and 2 (*r* = −.11, *p* = .33, *r* = −.13, *p* = .29, respectively) but there was a significant negative correlation between the IPSS questionnaire score and testosterone values in group 3 (*r* = −.29, *p* = .03).

## 4 DISCUSSION

In this study, we evaluated the relationship between benign prostatic hypertrophy's (BPH) symptoms and atrial fibrillation (AF) predictors; atrial electromechanical delay time (AEMD) and the P‐wave dispersion (PWD). We found that there is a significant positive correlation between BPH symptoms severity and these parameters which suggests that with more symptomatic BPH there is an increased risk of AF development.

Among old males with BPH, there are higher rates of cardiovascular (CVS) mortality and morbidity than their peers.^2^ Many mechanisms have been explored trying to explain this high mortality and morbidity. For example, these patients usually suffer from night symptoms, and this may lead to deteriorations of sleep quality and hence there will be disturbances in blood pressure regulation and impaired circadian blood pressure cycle. As a result, there will be impairment of the physiological event called dipping, which is manifested by a drop in nocturnal blood pressure detected in the normal population, and this may explain the increased rates of CVS events in BPH patients^16^ On the other hand, studies showed that there is an increased prevalence of coronary artery disease among patients with BPH which may be related to the androgen‐dependent smooth muscle proliferation that plays role in the development of both atherosclerosis and prostatic hypertrophy.^17^ The prevalence of AF was also found to be increased in BPH patients^5^ which is one of the most important clinical conditions that can contribute to the increased rates of CVS events.^10^, ^18^ Among this population, the early prediction of AF can be vital in decreasing the rates of related systemic thromboembolism, stroke, and other morbid and mortal CVS events. Besides, predicting AF in these patients will significantly reduce the cost burden of their clinical condition.^19^

In our study, we aimed to find methods for early detection of AF in BPH patients using the electrophysiological parameters AEMD and PWD which were found to be significantly longer in patients with new‐onset AF compared to the normal population in many clinical trials.^11^, ^12^ In one study, an increased incidence of postoperative AF was found in a group of patients with higher AEMD than the group with lower AEMD durations.^20^ Also, in studies evaluating the relationship between PWD and AF, prolonged PWD which is a marker of atrial remodeling was associated with episodes of paroxysmal AF, as well as with the recurrence of AF after conversion to sinus rhythm.^21^, ^22^ All these studies demonstrate that both AEMD and PWD can be very useful in predicting the risk of developing AF. To correlate these electrophysiological parameters with BPH symptoms, we used the IPSS questionnaire to identify symptoms severity. In our study results, there were significant positive correlation between IPSS scores and both AEMD and PWD in all patients (for AEMD *r* = .29, *p* = .013 and for PWD *r* = .27, *p* = .017), this correlation was most prominent in the group with severe symptoms and highest IPSS (for AEMD *r* = .33, *p* = .01 and for PWD *r* = .3, *p* = .02). According to these results, BPH patients with higher IPSS score had a more prolonged AEMD and PWD duration compared to those with lower scores suggesting that patients with more severe symptoms may be at higher risk of developing AF. To understand the direct relationship between clinical AF and BPH symptoms, we checked the patients' long‐term medical records from our national registry and contacted the patients 6 month after the study period to look for symptomatic AF attacks. It is noteworthy to mention that the results of this follow‐up supported our main study results as AF was detected in seven patients among the third group with the highest IPSS scores and only in three patients in total among the other two groups with lower IPSS scores.

The increased risk of AF in BPH patients can be explained through some common pathophysiological factors that can contribute to the development of both conditions. For example, chronic inflammation which is considered as an important etiological factor in the development of BPH was also found to play role in developing AF.^6^, ^23^ A study of stroke patients with new onset or episodes of paroxysmal AF has shown that increased serum inflammatory markers was associated with prolonged PWD and thus supporting the role of inflammation in developing AF.^24^ Another important factor is the role of testosterone in BPH disease as studies have shown that low levels of serum testosterone are directly related to the development and the progress of prostatic hyperplasia aggravating lower urinary tract symptoms.^8^, ^23^ On the other hand, the FINRISK97 study showed that low testosterone levels have a predictive value for future atrial fibrillation development in men.^9^

In our study, there were significant differences between the three groups in terms of inflammatory markers C‐reactive protein (CRP) and fibrinogen serum levels (*p* < .01 and *p* < .01, respectively). There were also a significant positive correlation between IPSS score and CRP values in groups 2 and 3 (*r* = .22, *p* = .049 and *r* = .29, *p* = .01, respectively) and a significant positive correlation between the IPSS score and fibrinogen values in group 3 (*r* = .25, *p* = .02). These results suggest a direct correlation between BPH symptoms severity and the status of inflammation in the body. Testosterone levels were low in all patient groups but there were significant differences between the groups, with the group of highest IPSS score having the lowest testosterone levels (*p* < .01). There were also a significant negative correlation between the IPSS score and testosterone values in group 3 with the highest IPSS score (*r* = −.29, *p* = .03), suggesting that the severity of BPH symptoms is also directly related to testosterone levels. In summary, all these results show that in patients with more severe BPH symptoms there are higher status of inflammation, lower levels of testosterone and higher risk of developing AF. In the light of all mentioned above, we can conclude that periodic evaluation of patients with BPH can be helpful not only from urological aspect, but also in the early prediction of possible serious cardiovascular morbidity and mortality.

The main limitation of this study is the small number of participants, the larger number of patients involved could have provided us with healthier data. Another important point is the lack of a long‐term cardiac rhythm monitoring using a Holter device or an implantable loop recorder which could have helped in identifying cases of clinical AF for a better interpretation of the study results.

## CONFLICT OF INTEREST

The authors declare that there is no conflict of interest regarding the publication of this article.
